# Limb Ischemia after Heart Transplantation: An Unusual Case of Tissue Embolism

**Published:** 2017-04

**Authors:** Seyed Mohsen Mirhosseini, Ali Sanjari Moghaddam

**Affiliations:** 1 Chronic Respiratory Disease Research Center, National Research Institute of Tuberculosis and Lung Diseases, Shahid Beheshti University of Medical Sciences, Tehran, Iran.; 2 School of Medicine, Shahid Beheshti University of Medical Sciences, Tehran, Iran.

**Keywords:** *Heart transplantation*, *Cardiac surgical procedures*, *Complications*, *Acinetobacter baumannii*

## Abstract

Major complications of heart transplantation include graft rejection, infection, graft arteriosclerosis, malignancy, and drug toxicity. Among these complications, infections and thrombophilic disorders are of particular interest owing to their major contribution to morbidity and mortality among heart transplantation patients. Thrombophilic disorders are caused by imbalance between hypercoagulation and fibrinolytic states. In this report, we describe a 43-year-old man who had unusual complications of heart transplantation. We presume that the unusual postoperative complications of the patient might have been caused by a faulty surgical procedure, improper use of anticoagulant agents, and incomplete prophylaxis for infections. During the postoperative period, the patient suffered arterial obstruction three times, for which he underwent clot removal via embolectomy. In addition to arterial obstruction, the patient had a mobile mass in the left atrium that was removed by open cardiac surgery. The frozen sample of the cardiac mass was positive for Acinetobacter baumannii. After 7 days of observation in the hospital and proper antibiotic regimen, the patient was sent home with no additional complaints and normal physical examination. We conclude that in heart transplantation patients, the precise performance of the surgical procedure, postoperative care, and early removal of the embolus might reduce morbidities and mortality due to thrombophilic disorders.

## Introduction

Post heart transplantation complications such as graft rejection, infection, graft arteriosclerosis, malignancy, and drug toxicity remain the major cause of mortality and morbidity around the world.^1^^, ^^2^ Infection is the leading cause of death after heart transplantation.^3^ It has been demonstrated that 31% of the patients experience post-transplantation infection, mainly bacterial and viral.^3^ In addition to infection, thrombophilic disorders, which lead to postoperative thromboembolic complications, are considered as another major cause of morbidity and mortality in cardiac surgeries.^4^ Another major complication is hypercoagulation, resulting from the stress response to surgery and the contact between the circulating blood and the cardiopulmonary bypass circuit, though this complication is managed with strong anticoagulants and/or fibrinolytics administration.

In this paper, we report unique and very unusual complications following cardiac transplantation for a 43-year-old man.

## Case Report

A 43-year-old man with a history of dilated cardiomyopathy (functional class IV) who poorly responded to full-dose treatment for heart failure was admitted to Masih-Daneshvari Hospital for heart transplantation in August 2012. The mean pulmonary artery pressure (PAP) of the patient was 24 mmHg (peak PAP = 35 mmHg), and echocardiography showed an ejection fraction of 15%. 

The donor was a previously healthy 22-year-old man who died of traumatic brain injury. The evaluation of the donor indicated a compatible blood group with the patient (both B+) with acceptable body surface area.

The transplantation was performed via orthotopic bicaval heart transplantation technique. A patent foramen ovale (PFO) was found in the heart of the donor in the operating room, which was closed up. The duration of clamping ischemia was 130 minutes. The heart transplantation procedure was performed properly with no unusual complication, and the patient was transferred to the intensive care unit (ICU) and was extubated after 2 days.

Ten days after heart transplantation, the patient complained of pain in the upper left limb, ague, and sweating. Initial assessment indicated that he was febrile with tachycardia. Examination of the extremities revealed coldness with decreased sensation in the left upper limb. Paraclinical assessments showed leukocytosis (white blood cell = 35000/mm^3^ and neutrophil = 87.4%). Doppler ultrasound of the left upper limb was performed to rule out arterial occlusion. In Doppler, an obstruction due to clot formation in the brachial artery, 2 cm away from the axillary fold, was confirmed. 

With regard to the urgency of the condition, the patient was prepared for embolectomy of the brachial artery. A Fogarty arterial embolectomy catheter was utilized, and a clot and some tissue-like material were removed. The normal blood flow of the brachial artery was reestablished. After a few minutes, the pulses of the brachial and radial arteries disappeared once again and new arteriotomy and embolectomy were performed at the same place. Again, the same material was removed, and normal blood flow was returned. A day after the procedure, transesophageal echocardiography (TEE) was carried out and showed multiple hypoechoic and mobile masses in the left atrium (LA) above the left atrium auricle and within the right atrium (RA) just adjacent to the inferior venae cava ostium (cardiac suture lines). Moreover, a 2 × 2 cm loculation in the posterior aortic wall in the transverse pericardial sinus was seen. All of the valves were normal. Plain brain computed tomographic (CT) scan was performed to assess the probable presence of cerebral embolism and it had no apparent abnormality. In addition, chest spiral CT was conducted to investigate the possibility of pulmonary embolism and it demonstrated no active parenchymal infiltration and no pleural effusion. 

Given the TEE result, the patient was prepared for emergent surgery to remove the heart masses. Both venae cavae and aorta were cannulated. Within an aortic clamping time of 25 minutes, the RA was opened and the masses on the suture line and near the inferior vena cava were removed. The tricuspid valve was normal. The septum was assessed for the presence of the PFO and it was completely closed. Then the septum was opened, and the mobile masses were removed at different points in the LA. The mitral valve was also normal. All spaces in the LA and RA were irrigated with saline. The septum was closed in two layers and air was drawn. Finally, TEE was performed and no lesion was seen. During the surgery because of coldness and disappearance of the radial pulse in the left upper limb, the Fogarty catheter was used in the previous site of arteriotomy once again and a large clot was removed from the brachial, radial, and ulnar arteries. 

The patient was transferred to the ICU with a good hemodynamic condition. Frozen samples showed an exudate fluid with a great number of white blood cells, which favored infection. Culture of the samples was positive for *Acinetobacter baumannii*. After 7 days of observation in hospital and proper antibiotic regimen, the patient was discharged home with no additional complaints and normal physical examination. A 3-month follow-up showed normal good condition of the patient without the recurrence of the symptoms.

## Discussion

In this study, we describe a 43-year-old man who underwent heart transplantation and had unusual postoperative complications, including three incidents of arterial occlusion and mobile heart masses, which were managed via multiple embolectomy and removal of the mobile masses in the LA through open cardiac surgery. 

Many studies have shown that the common complications of cardiac transplantation include infection, rejection, accelerated coronary artery atherosclerosis, and lymphoproliferative diseases^1^^, ^^2^ as well as thromboembolic disorders.^5^^, ^^6^ Acute limb ischemia due to acute arterial occlusion is an infrequent finding in patients with recognized peripheral artery disease, except in those undergoing surgical or endovascular revascularization with a conduit, graft, or stent. Acute limb ischemia usually happens in middle-aged and older people but can disturb the life of younger population when unusual clinical events such as paradoxical embolism, intracardiac masses, and endocarditis, or hypercoagulable syndromes affect the arterial circulation.^7^ Acute nontraumatic ischemia of the upper extremity, which is almost always caused by an embolus, is more uncommon than that of the lower extremity.^8^ Typically, acute arm ischemia accounts for 16.6% of the cases of acute ischemia of the extremities with an estimated incidence of 1.2 to 3.5 cases per 100000 people per year.^7^ The brachial and axillary arteries are the most common sites of arterial occlusion in the upper extremity and represent 85% of the cases of embolic occlusion. In a recent study of 65 patients with acute arm ischemia treated surgically over a period of 8 years, a cardioembolic source was identified in 41% of the patients, 17% of the events were recognized with an arterial source of embolism, and 28% of the cases were related to iatrogenic occlusion, mostly a result of cardiac catheterization.^7^ In our report, the cardiac mass was a potential source of arterial emboli.

In the setting of the embolism of the extremities, the ideal is achieved by the complete removal of the embolus with restoration of the circulation continuity.^9^ Despite the fact that rapid embolectomy with a balloon catheter is the best management of arterial emboli,^8^ the rate of the embolectomy procedures of the extremities appears to have fallen. Although the cause for this trend is not known, one possible explanation is the increasing prescription of anticoagulants after major interventions of the cardiovascular system.^10^

Tissue embolism, as an unusual complication of heart transplantation, has been addressed by a few studies.^11^^-^^13^ Air bubbles, thrombus fragments, bone marrow fragments, foreign bodies (e.g. cotton and wax), and fatty tissue fragments have been demonstrated as different materials of emboli.^13^ In a setting of embolism in a patient on anticoagulation medication (as occurred in our patient), it is very important to find the source and etiology of the embolism by thoroughly evaluating the heart in such a condition. In the present study, we found evidence of *Acinetobacter baumannii* infection in the cardiac masses. To the best of our knowledge, this case is the first one with this kind of infection and subsequent septic emboli following cardiac transplantation. 

## Conclusion

We presume that the frequent embolism in the extremities and infection may reflect intraoperative defects, unsterile condition, and inadequate prophylactic antibiotic therapy and dose of the anticoagulant. To avoid these complications, we recommend precise performance of the operation, proper prophylactic antibiotic therapy, and complete courses of anticoagulant agents.

## Notes:

***This paper should be cited as:*** Mirhosseini SM, Sanjari Moghaddam A. Limb Ischemia after Heart Transplantation: An Unusual Case of Tissue Embolism. J Teh Univ Heart Ctr 2017;12(2):92-94.
